# An Insight into the Endophytic Bacterial Community of Tomato after Spray Application of Propiconazole and Bacillus subtilis Strain NBRI-W9

**DOI:** 10.1128/spectrum.01186-22

**Published:** 2022-09-06

**Authors:** Udit Yadav, Nasreen Bano, Sumit Bag, Suchi Srivastava, Poonam C. Singh

**Affiliations:** a CSIR-National Botanical Research Institute, Lucknow, Uttar Pradesh, India; b Academy of Scientific and Innovative Research (AcSIR), Ghaziabad, Uttar Pradesh, India; University of Minnesota

**Keywords:** biofungicide, *Bacillus subtilis*, endophyte, propiconazole, tomato

## Abstract

Propiconazole (PCZ) is a commonly sprayed fungicide against fungal pathogens. Being systemic in action, it reaches subcellular layers and impacts the endophytes. Although PCZ is a fungicide, it is hypothesized to exert an inhibitory effect on the bacterial endophytes. Therefore, this study aims to get an insight into the perturbations caused by the systemically acting antifungal agents PCZ and Bacillus subtilis (W9) and the consequences thereof. The current study compared the 16S rRNA microbial diversity, abundance, and functions of the endophytic bacterial community of tomato in response to PCZ, W9, and PCZ+W9 application. The implications of these treatments on the development of bacterial speck disease by Pseudomonas syringae were also studied. The culturable endophyte population fluctuated after (bio)fungicide application and stabilized by 72 h. At 72 h, the endophyte population was ~3.6 × 10^3^ CFUg^−1^ in control and ~3.6 × 10^4^ in W9, ~3.0 × 10^2^ in PCZ, and ~5.3 × 10^3^ in PCZ+W9 treatment. A bacterial community analysis showed a higher relative abundance of *Bacillales*, *Burkholderiales*, *Rhizobiales*, *Pseudomonadales*, and *Actinomycetales* in the W9 treatment compared with that in the PCZ treatment and control. Phylogenetic investigation of communities by reconstruction of unobserved states (PICRUSt) analysis showed enhanced metabolic pathways related to secretion, stress, chemotaxis, and mineral nutrition in the W9 treatment. Disease severity was greater in PCZ than that in the W9 treatment. Disease severity on tomato plants showed strong negative correlations with *Sphingomonas* (r = −0.860) and *Janthinobacterium* (r = −0.810), indicating that the natural biocontrol communities are agents of plant resistance to diseases. Outcomes show that systemic chemicals are a potential threat to the nontarget endophytes and that plants became susceptible to disease on endophyte decline; this issue could be overcome by the application of microbial inoculums.

**IMPORTANCE** Endophytes are plant inhabitants acting as its extended genome. The present study highlights the importance of maintaining plant endophytes for sustainable disease resistance in plants. The impact of chemical fungicides and biofungicides was shown on tomato endophytes, in addition to their implications on plant susceptibility to bacterial speck disease. The observations point toward the deleterious effects of systemic pesticide application on endophyte niches that disrupt their diversity and functions compromising plant immunity.

## INTRODUCTION

Plants support a complex microecosystem comprising of distinct bacterial communities on or inside the plants. These microorganisms growing inside the plant tissues constitute the endophyte population. Endophytes can colonize both the intercellular and intracellular region of the plant tissue and exist in mutualism with the plant, affecting its survival and health (1, 2). The impact of bacterial endophytes on the plants may be direct or indirect, and their activities range from nitrogen fixation to plant growth promotion via plant hormone and enzyme synthesis (3). Bacterial endophytes produce plant growth hormones, such as indole acetic acid, gibberellic acid, and cytokinins (4). Besides PGP activities, biological control, induced resistance to phytopathogens, and stress tolerance mediated by the endophytes are also well documented (5–7). Thus, endophytes play an imperative role in maintaining plant health. Although the endophytes occupy protective niches inside plants, their population and diversity are bound to be influenced by various factors, including nutrient status, abiotic and biotic stress, and agrochemical application. Agrochemicals, such as fertilizers and pesticides, are used regularly on plants which affect the nontarget microorganisms disrupting a wide spectrum of activity in agroecosystems (8, 9). These agrochemicals affect the microbial diversity and induce shifts in the microbial community structure of soil, aquatic, and phyllosphere habitats (10–12). Therefore, knowledge of the potential effects of such agrochemicals and biological treatments on the taxonomic structure and functional properties of the endophytic microbiota will be important. Propiconazole (PCZ) belongs to the triazole group of systemic fungicides. The volume of triazole fungicide has doubled over the last 25 years, and its market value has increased more than four times, currently with about a 16% share of the global fungicide volume market, and has been increasing steadily since the 1990s (13). PCZ has been reported to affect nontarget organisms in soil and water (14–16). Being systemic in nature, PCZ starts functioning through entering the plant system; therefore, it is inevitable that PCZ will come in direct contact with the nontarget bacterial endophytes. The half-life of PCZ is estimated to be 5 to 6 days in plants (17); therefore, its maximum effect would be observed within a week of application. In the course of time, PCZ will exert its inhibitory activity on the target fungal pathogen(s) and toxicity on the nontarget endophytic bacterial microflora. PCZ has been reported to impart bacterial community changes and inhibit the growth of soil bacteria (18). Scant reports are available describing the effects of some fungicides on the fungal endophytes. However, there are no reports of PCZ effects on the bacterial endophytes. Since fungicides are currently a necessity for global food security and will be continued to be used (19), application of biological control agents along with integrated applications with chemical pesticides are being promoted to reduce the chemical inputs (20). These chemical and biological treatments could potentially affect taxonomic structures and functional properties of the endophytic microbiota, and deeper knowledge about this topic will help in perceiving and overcoming the plant’s susceptibility to biotic and abiotic stresses. Therefore, study is needed to understand their effect on the nontarget bacterial endophytes of the plants on which they are being sprayed and minimize the potential threats related to plant health, microbial diversity, and sustainability. Biological control and plant-growth-promoting agents, such as *Trichoderma* spp., Bacillus subtilis, and Pseudomonas fluorescence, are alternatives to chemical fungicides that are used extensively and widely. Like the chemicals, the spray application of biofungicides may also affect the overall diversity and structure of the nontarget microorganisms which may or may not be dominated by the inoculums. Since the use of biofungicides is increasing gradually in the global market and is estimated to reach 47,000 ton/year by 2024 (21), their impact on plant endophytes is also of importance, as they are the drivers of the future integrated and/or chemical-free management of fungal pathogens. Therefore, Bacillus subtilis strain NBRI-W9, known for its biocontrol and plant-growth-promoting activity (22), was used in the present study as the positive control.

Tomato (Lycopersicum esculentum) is one of the major fruit crops grown worldwide, and it is susceptible to a large spectrum of fungal pathogens; therefore, it is frequently sprayed by chemical fungicides. In extensive farming, triazoles are relatively inexpensive and effective; thousands of tons of triazole fungicides are thus used for crop protection every year (23). The impact of commercial fungicides and biocontrol agents on the structure and function of endophytic microbiota is unknown in tomatoes.

It is hypothesized that externally applied systemic chemicals and bioinoculants will affect the endophytic bacterial communities that will impact the overall homeostasis and the plant’s susceptibility to a pathogen. Therefore, the objective of the present study was to investigate the effect of a systemic fungicide, propiconazole (PCZ), and a biocontrol agent, Bacillus subtilis NBRI-W9 (W9), on the taxonomic structure and functional properties of endophytic microbial communities present in tomato leaves. Furthermore, plant susceptibility to disease was assessed in the presence of PCZ and W9 against a bacterial pathogen, P. syringae, which is the causal agent of bacterial speck of tomato (24). The rationale behind choosing the bacterial pathogen P. syringae, instead of a fungus, was to eliminate the direct role of the (bio)fungicide (PCZ and W9), which is to inhibit fungi and not bacteria, and instead to assess only the role of plant immunity/susceptibility to infections which follows the fungicide and biofungicide application.

## RESULTS

### Compatibility of propiconazole (PCZ) with Bacillus subtilis NBRI-W9.

**FIG 1:**
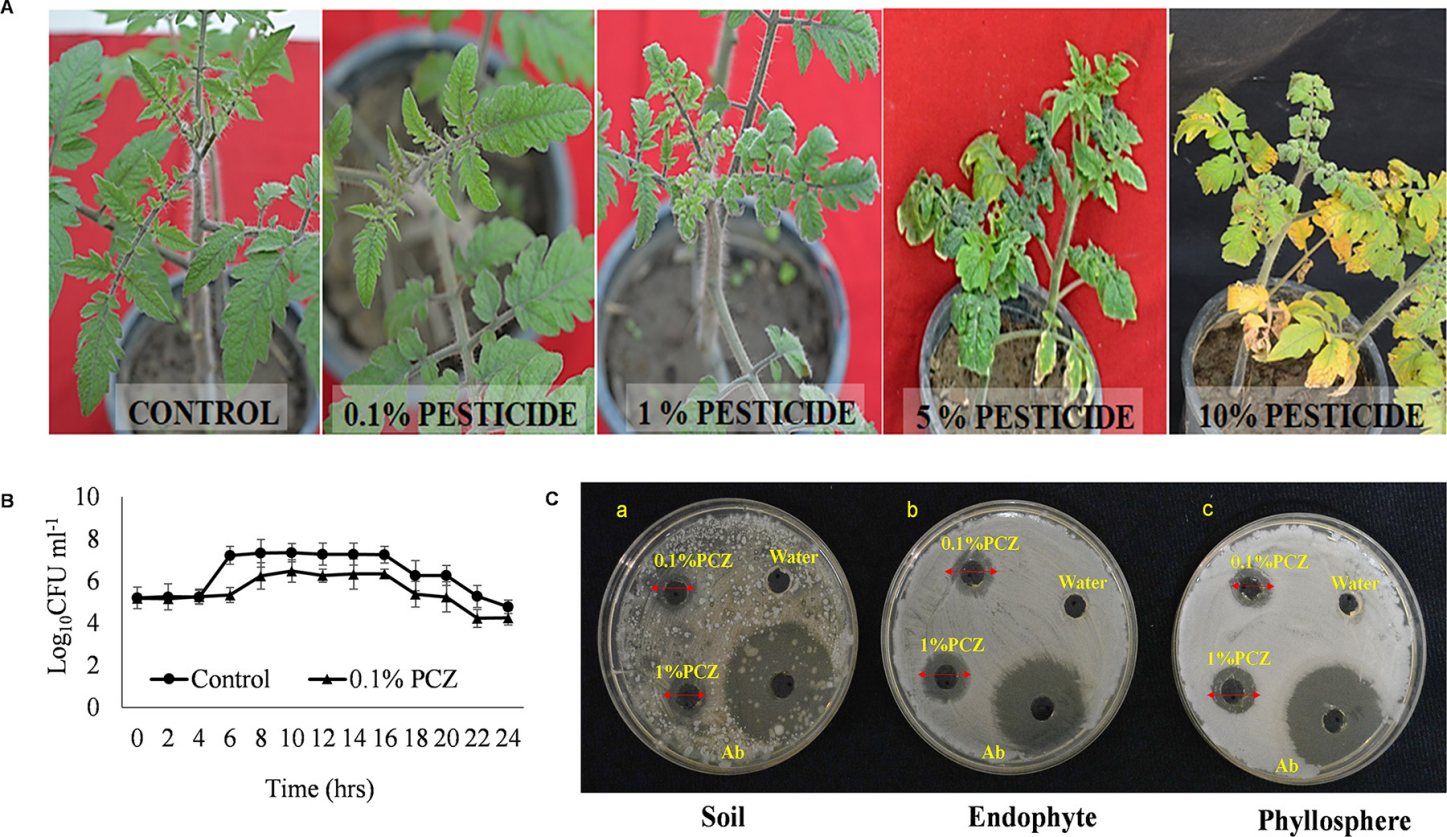
(A) Effect of propiconazole (PCZ) after spraying different strengths (10%, 5%, 1%, and 0.1%) of a commercial formulation on 1-month-old tomato plants. (B) Growth curve of NBRI-W9 with PCZ (0.1%). (C) Inhibitory effect of PCZ (0.1% and 1%) on the bacterial community of the tomato rhizosphere (a), leaf phyllosphere (b), and leaf endophyte (c); negative control, water; positive control, 100 mg ml-1 streptomycin (Ab).

The agar well diffusion assay with NBRI-W9 was performed using two different concentrations of PCZ, i.e., 0.1% and 1%, to check their compatibility. The results showed that B. subtilis NBRI-W9 was compatible with PCZ, as there was no zone of inhibition with both the concentrations. A zone of inhibition was formed (7.0 mm ± 0.02) with streptomycin which was used as the positive control (Fig. 1B).

### Effect of PCZ on the nontarget natural bacterial population of tomato rhizospheric soil, phyllosphere, and endophytes (*in vitro*).

The effect of PCZ on the nontarget bacterial population was studied using the natural heterogeneous sources of the bacteria, such as rhizospheric soil, tomato phyllosphere (unsterilized leaf), and tomato endophytes (surface sterilized leaf). An inhibitory effect of PCZ (0.1% and 1%) was observed on the growth of bacterial populations from soil, phyllosphere, and endophytic microflora of tomato; however, it was nonbactericidal. The PCZ showed a maximum inhibition of the tomato endophytes followed by the phyllosphere and soil (Table 1 and Fig. 1C).

**TABLE 1:** Inhibitory effect of propiconazole on the bacterial population of soil, phyllosphere, and endophytes of tomato

Treatment	Zone of inhibition (mm) of^*a*^:		
	Control	0.1% PCZ	1.0% PCZ
Tomato rhizosphere soil	0.0 ± 0.00	15.00 ± 0.47	16.33 ± 0.27
Tomato phyllosphere	0.0 ± 0.00	13.66 ± 0.27	16.66 ± 0.27
Tomato endophytes	0.0 ± 0.00	14.56 ± 0.24	20.00 ± 0.21

a Each value is mean of three replicates (mean ± SE).

### Effect of propiconazole on culturable endophytic bacterial population (*in vivo*).

**FIG 2:**
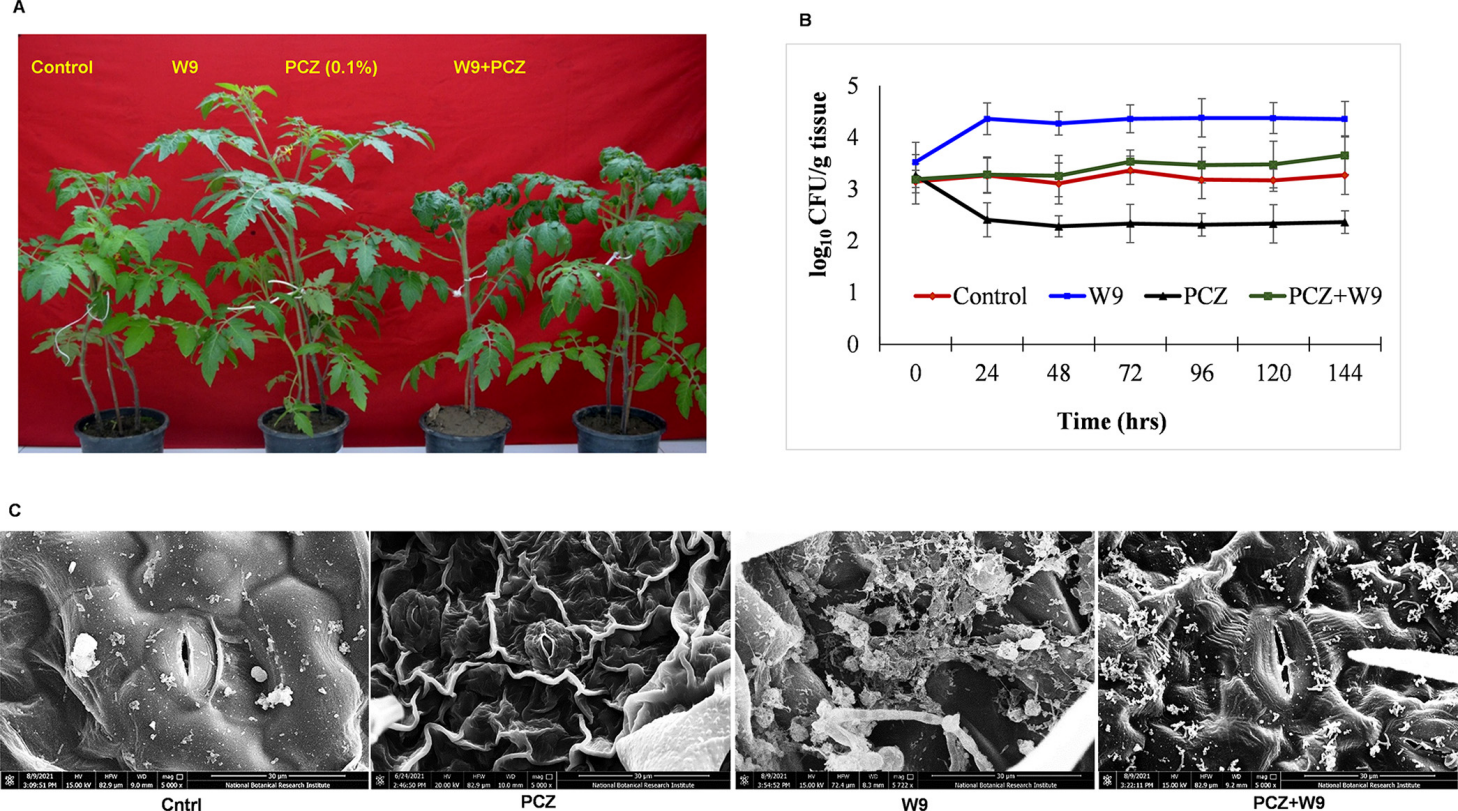
(A) Tomato plants treated with propiconazole (PCZ), B. subtilis (W9), and PCZ+W9. (B) Effect of PCZ and W9 application on putative endophyte population over a period of 144 h. (C) Scanning electron microscopic (SEM) images of tomato phyllosphere showing the effect of PCZ and W9 on the bacterial microflora (magnification, ~5,000×).

The effect of PCZ on the culturable endophytic bacterial population was determined in the tomato using 0.1% PCZ which is a nonphytotoxic and recommended dose on tomato leaves (Fig. 1A). In comparison to the 0.1% PCZ concentration, the 1%, 5%, and 10% sprays show phytotoxic effects (Fig. 1A). The bacterial endophytic population determined over a period of 144 h shows a significant loss of bacteria on PCZ application, while the populations increased in the presence of W9. The integrated application of PCZ and W9 decreased the impact of PCZ (Fig. 2B). The impact in terms of the bacterial CFU of surface-sterilized leaf tissue was taken from day 0 to the 21st day of the treatment. Population fluctuations were observed up to 72 h, after which it was stabilized. In control plants, the endophyte population was 4.14 ± 0.21 log_10_ CFU/g tissue and remained nearly constant for at least 144 h (Fig. 2B). Application of PCZ decreased the population from 4.26 ± 0.32 to 3.40 ± 0.30 log_10_ CFU g^−1^ in 24 to 48 h, 2.33 ± 0.27 log_10_ CFU g^−1^ in 72 h and remained constant thereafter (Fig. 2B). Thus, compared with the control plants, a persisting loss of the endophytic population by approximately 50% was observed after PCZ application. NBRI-W9 application increased the endophytes from 4.52 ± 0.22 to 5.36 ± 0.23 log_10_ CFU g^−1^ after 24 to 48 h and to 6.36 ± 0.36 by 72 h, and it remained constant thereafter. In the combined application of PCZ and W9, the population remained stable as in the control plants. From these results, it was inferred that the endophytic population was susceptible to PCZ and responded positively to W9 spray. The impact of the sprays was fast acting and stable, and 72 h could be the discriminating time interval for further study. Therefore, 72 h and a 0.1% PCZ concentration were selected as the tomato leaf sampling parameters for the microbial community analysis (16S rRNA) and other studies.

### Scanning electron microscopy of the phyllosphere microflora.

Scanning electron microscopy (SEM) of leaf samples at 72 h postinoculation (hpi) revealed some structural distortion on the leaf surface. A reduction in numbers and a morphologically different phyllosphere population compared with control leaf were also found (Fig. 2C, PCZ). The NBRI-W9-treated leaf surface showed higher bacteria in small aggregates resembling biofilm (Fig. 2C, W9). The PCZ+W9 micrograph showed that NBRI-W9 decreased the structural distortions and improved the phyllosphere microflora showing a morphologically distinct mixed bacterial population (Fig. 2C, PCZ+W9).

### Sequencing and quality control.

A total of 2,188,364, 2,502,524, 2,285,168, and 2,357,858 reads were obtained for control, W9, PCZ, and PCZ+W9, respectively. The quality-trimmed reads against the chloroplast genomes were mapped. Total chloroplast reads were 1,312 (0.08%), 639 (0.03%), 1,060 (0.06%), and 588 (0.03%) in control, W9, PCZ, and PCZ+W9, respectively. The average GC content and read lengths for these four samples were 53.25% and 251 bp, respectively.

### Bacterial diversity indices and community structure.

**FIG 3:**
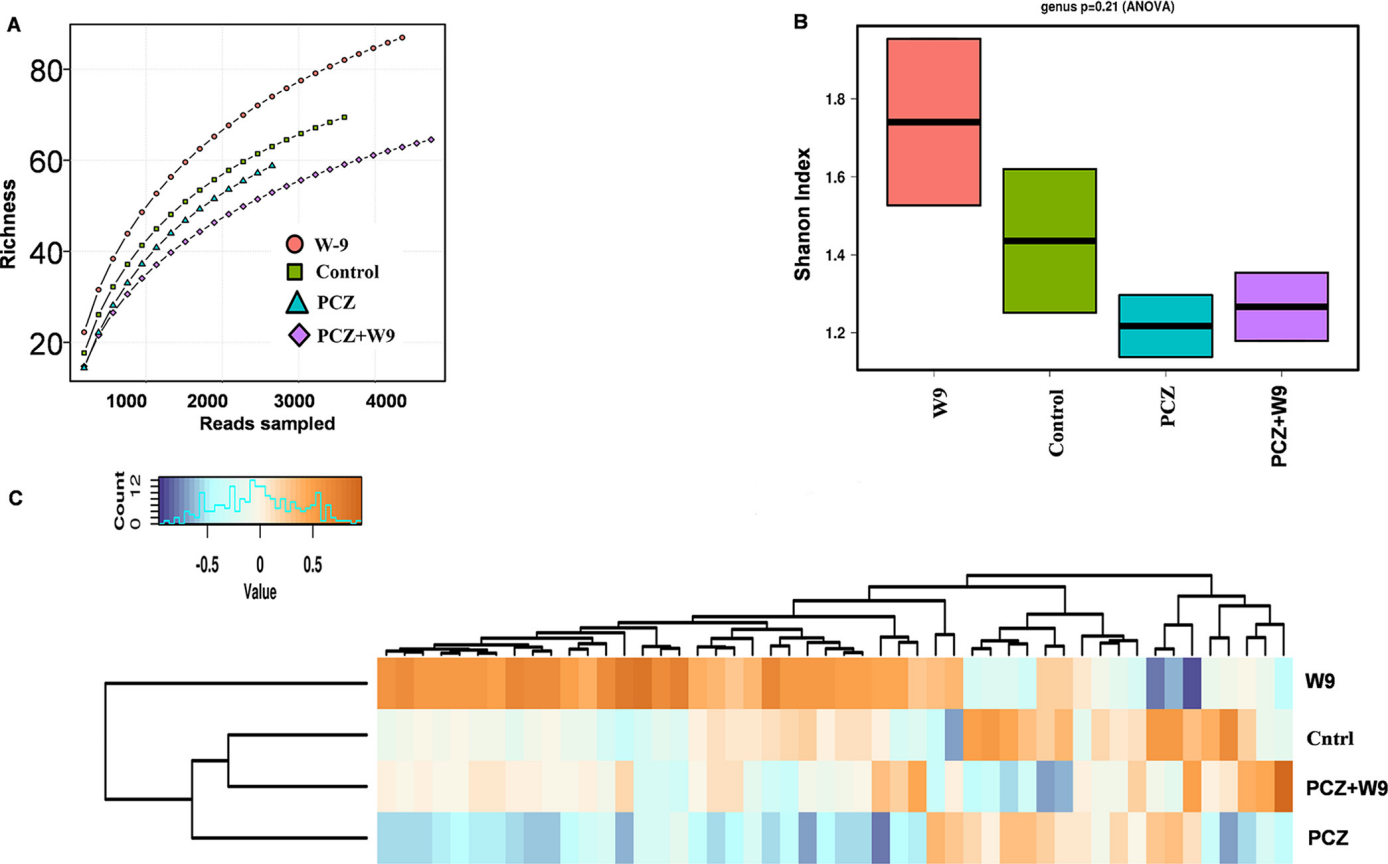
Endophytic bacterial diversity in tomato leaves treated with propiconazole and B. subtilis NBRI-W9. (A) Rarefaction curve showing the species richness, namely, alpha diversity, among the treatments. (B) Shannon diversity index. (C) Analysis of hierarchal clustering across different treatments.

**FIG 4:**
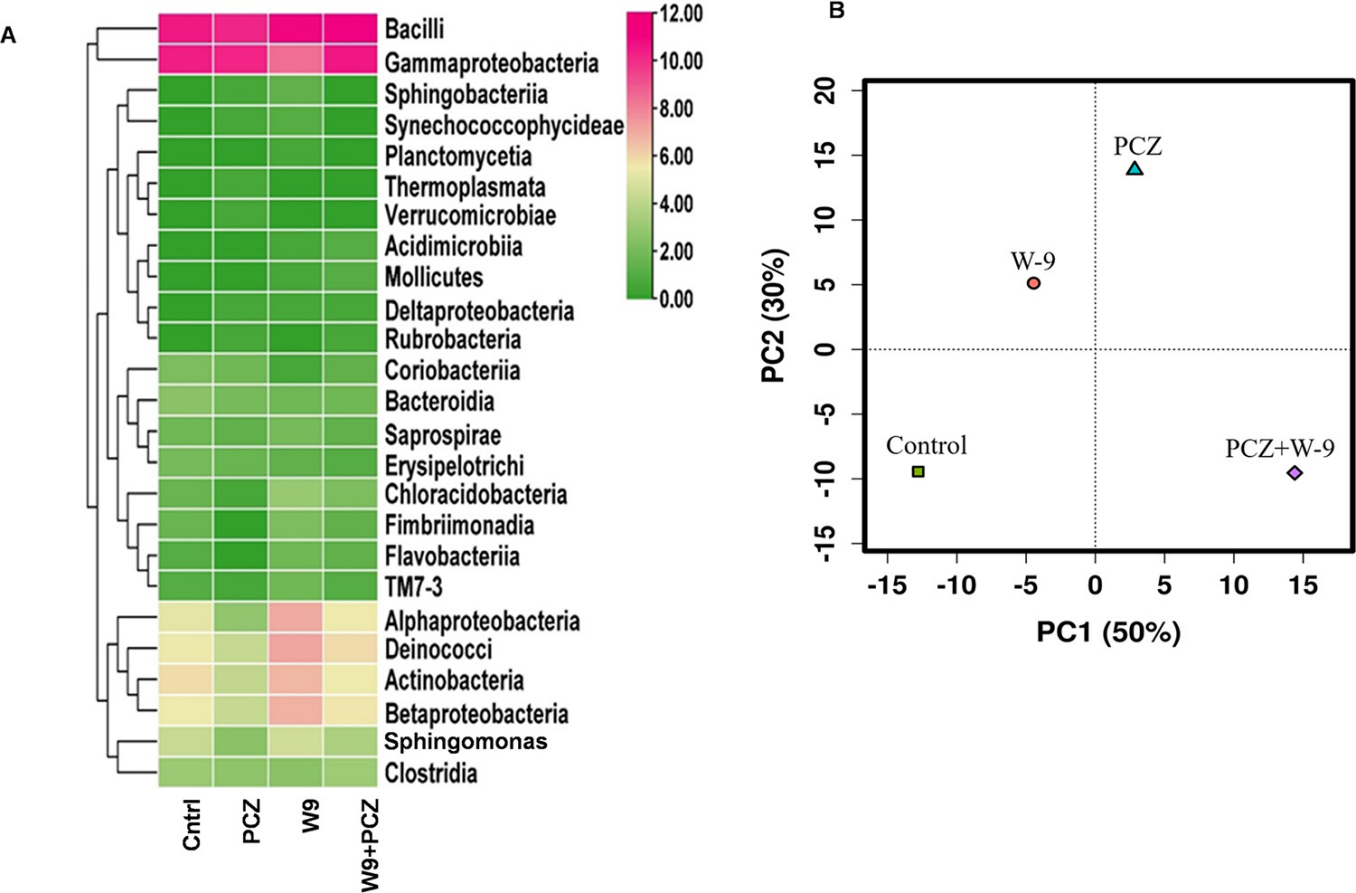
(A) Tomato endophytic bacterial diversity based on relative abundance at class level. (A) Heat map. (B) Principal-component analysis (PCA) showing beta diversity of the microbial communities based on operational taxonomic units (OTUs).

The effective sequences were clustered into operational taxonomic units (OTUs) using QIIME. A total of 250 endophytic bacterial isolates were identified spanning 130 bacterial genera according to the genetic differences from the 4 different leaf samples. The rarefaction curve showing the taxonomy indicates that the data contain sufficient sequence depth to ascertain the full bacterial diversity (Fig. 3A). The absence of a plateau in the rarefaction curve indicates more species are anticipated to be discovered. The species richness and diversity differed significantly among the four samples as observed from Shannon diversity index (Fig. 3B). The bacterial diversity was highest in the W9 sample (H index, 1.73) and lowest in the PCZ sample (H index, 1.21) (Fig. 3B). Furthermore, the principal-component analysis (PCA) plot analysis of the OTUs separated the treatments in different coordinates (Fig. 4B). On the PC1 scale, the W9-treated leaf was closer to the control than the PCZ- and PCZ+W9-treated leaf sample, which indicated that the control and W9-treated leaf had similar endophytic communities (Fig. 4B). In contrast, the control sample was located at a considerable distance from those of the PCZ-treated and PCZ+W9-treated leaf on the PCA plot suggesting that the fungicide had a significant effect on the endophytic bacterial community in the tomato leaf. Thus, from the PCA plot, it was concluded that PCZ and NBRI-W9 both were responsible for the shift in the microbial diversity, albeit in the opposite direction. The principal components on scale 1 were responsible for approximately 50% variability and separated the control and W9+PCZ treatment but occurred on the same plane on component 2. The 30% variability on scale 2 separated control and PCZ most widely. The PCA plot separation is further reflected from the hierarchical clustering across the different variables within the four treatments showing that microbiomes in NBRI-W9 did not cluster with other samples, while control and PCZ+W9 clustered together and away from PCZ and W9 (Fig. 3C). The heat map of the top 25 abundant microbial classes shows almost a similar pattern in control and PCZ+W9 treatments, while PCZ-and W9-treated samples show considerable differences in the relative abundances of the bacterial community at class level (Fig. 4A).

### Taxonomic composition and annotation of bacterial communities.

**FIG 5:**
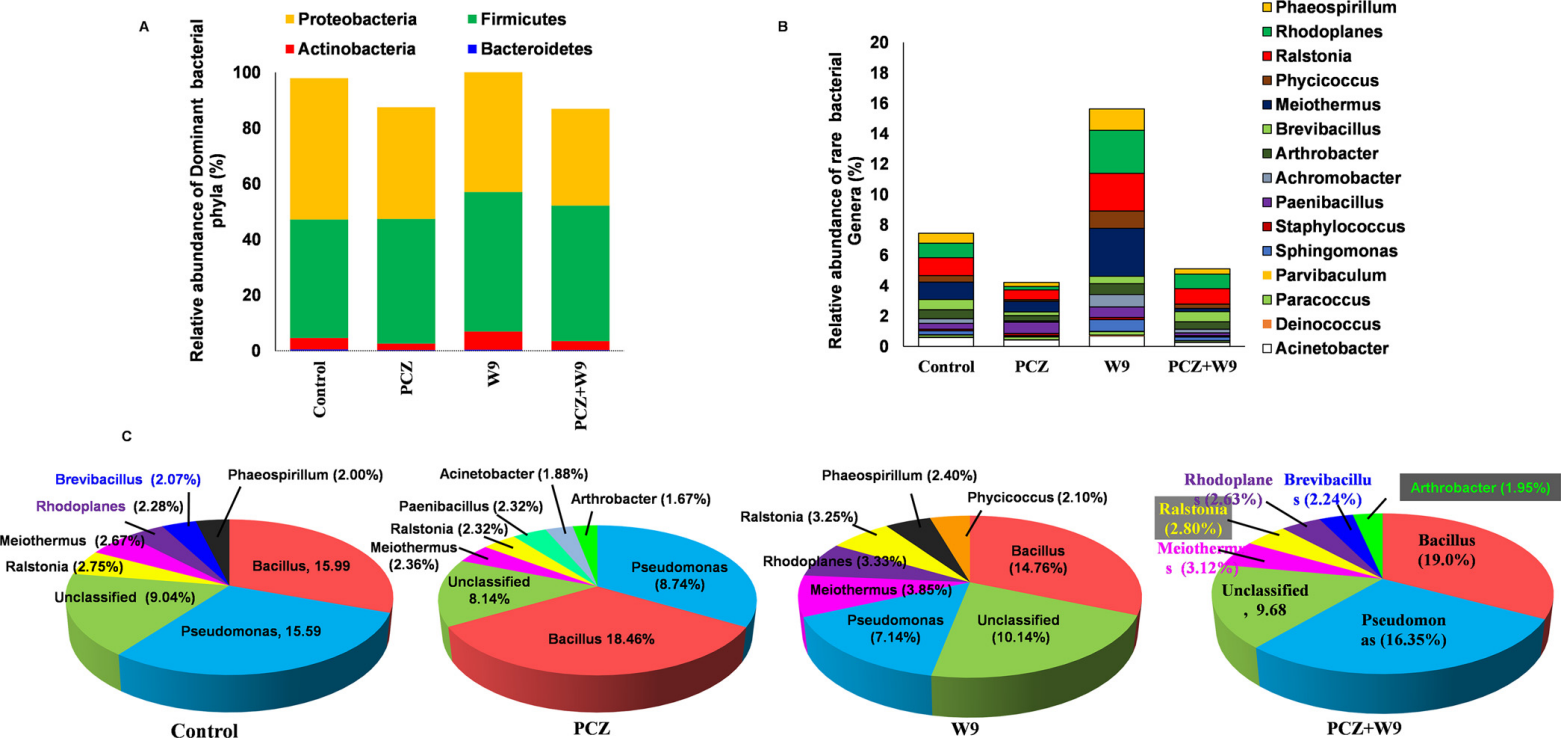
(A) Endophytic bacterial diversity based on relative abundance at the phylum level. (B) Relative abundance of top 15 genera at the genus level. (C) Genera showing >1.5% relative abundance.

A shift in the bacterial diversity was clearly detected in the tomato leaf endosphere (Fig. 5). Overall, the phyla *Firmicutes* and *Alphaproteobacteria* dominated the endophytic bacterial community in all the treatments followed by *Gammaproteobacteria* members, including *Bacteroidia*, *Fibrobacteria*, and *Actinobacteria* (Fig. 5A). The relative abundance of bacterial phyla varied in the control and PCZ, W9, and PCZ+W9 treatments. In particular, the relative abundances of class *Actinobacteria*, *Proteobacteria*, *Flavobacteria*, *Deinococci*, and *Chloracidobacteria* were significantly higher in W9 than those in the control, PCZ, and PCZ+W9 (Fig. 4A). Considering the control, PCZ-treated leaves showed a lesser relative abundance of the top 15 dominant bacteria (Fig. 5B). The relative abundance of the dominant genera was affected by PCZ treatments (Fig. 5B). However, W9 treatment showed a greater abundance of dominant genera followed by control and PCZ+W9 (Fig. 5B). The relative abundance of bacterial phyla *Firmicutes* (49.8%), *Alphaproteobacteria* (25.56%), *Gammaproteobacteria* (17.04%), *Betaproteobacteria* (5.28%) and *Actinobacteria* (5.62%) was significantly increased in W9 treatment compared with that in the control, while the PCZ and PCZ+W9 treatment showed less abundant diversity (Fig. 5A). A heat map shows the abundance of the top 25 microbial classes in which an almost similar pattern in the control and PCZ+W9 treatment were observed, while PCZ- and W9-treated samples show differences in relative abundance of the bacterial community at the genus level (Fig. 4A). The Fig. 5C shows a pie diagram of the genera showing more than 1.5% of relative abundance. The application of PCZ resulted in the dominance of *Bacillus* (18.46%) compared with the control (15.59%); W9 resulted in the dominance of unclassified bacteria (10.14%), *Meiothermus* (3.85%), *Ralstonia* (3.33%), and *Phycicoccus* (2.1%) compared with abundances of 9.04%, 2.67%, 2.75%, and 0.4%, respectively, in the control. Interestingly, NBR-W9 (B. subtilis) application reduced the overall *Bacillus* abundance (14.76%) and was minimal compared with all other treatments. PCZ+W9 treatment enhanced the dominance of Pseudomonas (16.35%) and *Bacillus* (19%) compared with their abundances of 15.59% and 15.99%, respectively, in the control. Interestingly, the relative abundance of Pseudomonas which is one of the main biocontrol agents in the agroecosystem was reduced to nearly half in PCZ (8.74%) and W9 (7.14%) applications. *Arthrobacter* was the common genera present in PCZ and PCZ+W9, while *Bacillus* was considerably increased in these two treatments (Fig. 5C).

At the order level, the relative abundances of *Bacillales* (48.29%), *Burkholderiales* (5.08%), *Rhizobiales* (7.6%), *Pseudomonadales* (12.3%), and *Actinomycetales* (5.46%) were found higher in W9, while in PCZ treatment, decreased abundances of *Burkholderiales* (0.74%), *Rhizobiales* (2.69%), *Pseudomonadales* (5.08%), and *Actinomycetales* (2.52%) were observed compared with those in the control. An increased relative abundance of order *Bacillales* (42.44%) was observed in PCZ treatment as compared with the control (40.56%). PCZ+W9 treatment also showed an increased relative abundance in the abovementioned orders of bacteria compared with the control sample.

### Functional analysis predicted by PICRUSt.

**FIG 6:**
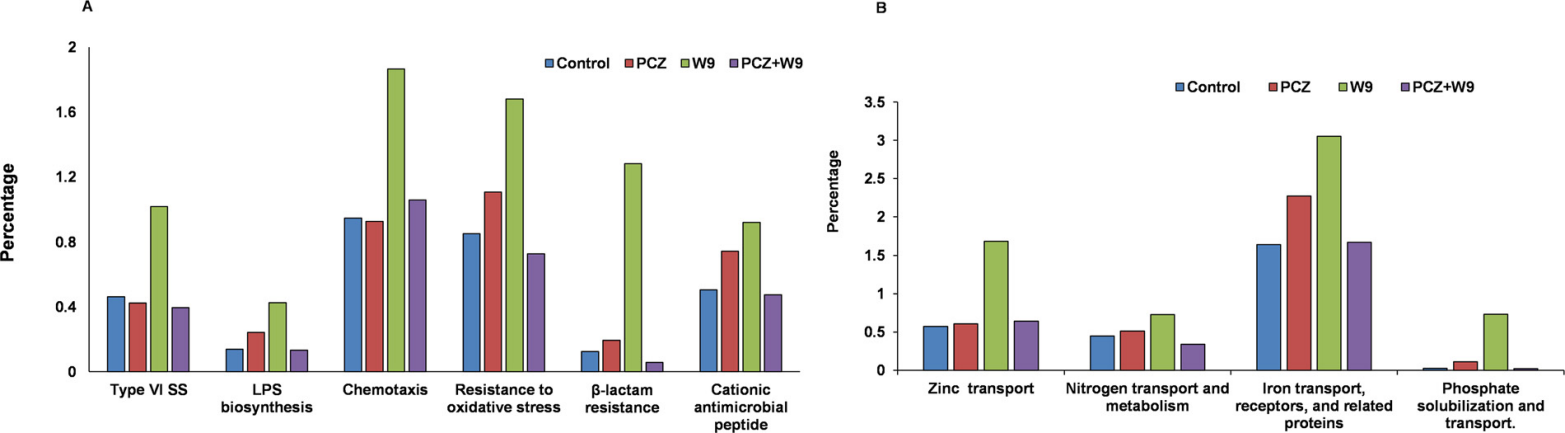
PICRUSt predictions of the functional composition of the endophytic microbiome. (A) Relative abundance of functions related to secretion, biotic and abiotic stress, and chemotaxis. (B) Mineral nutrition-related functions.

The phylogenetic investigation of communities by reconstruction of unobserved states (PICRUSt) analysis was used to explore the different metabolic potentials of the endophytic microbiota. The metabolic pathways, including metabolism, genetic information processing, environmental information processing, cellular processes, organismal systems, and human diseases, were all detected in the endophytic bacterial profiles. For endophytic bacteria, PICRUSt analysis revealed that type VI secretion system (T6SS) metabolic pathway gene family (1.18%) was increased in the W9 treatment compared with that of the control (0.4%) and PCZ (0.8%) and PCZ+W9 (0.3%) treatments. The chemotaxis-related metabolic pathway gene family increased in the W9 (1.9%)-treated plant compared with that in the control (0.9%) and PCZ (1.6%) and PCZ+W9 (0.9%) treatment. In the W9 treatment, resistance against oxidative stress metabolic pathways (1.7%), osmoprotectants, and compatible solute (e.g., proline, glycine betaine, trehalose, and spermidine) synthesis pathway gene family was increased compared with that in the control (0.8%), PCZ (1.0%), and PCZ+W9 (0.9%) (Fig. 6A). W9 treatment also increased the transporter gene family of minerals like Zn (0.9%), N (0.9%), Fe (3.4%), and P (0.4%) compared with the control and PCZ and PCZ+W9 treatments (Fig. 6B).

### Protective role of endophytes and plant response against Pseudomonas syringae in tomato plants.

**FIG 7:**
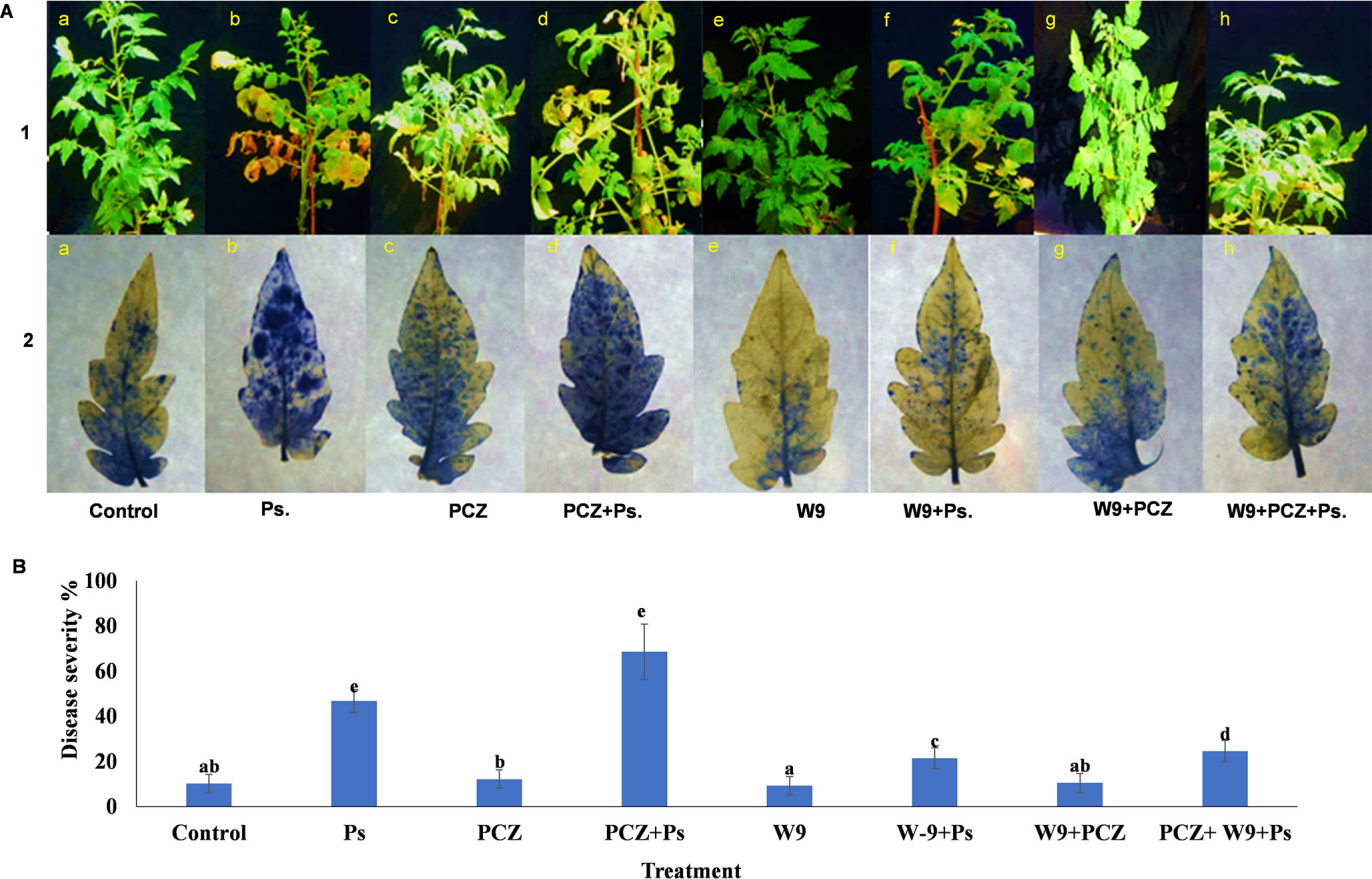
(A) Tomato plants (1) and their NBT stained leaves (2) showing various degrees of disease severity after 21 days of P. syringae inoculation in response to PCZ and W9 treatments. (B) Disease severity is expressed as the proportion of leaflet area that was necrotic. The mean scores and SE of four replicates (four leaves/replicate) are presented for each sample. Different letters indicate significant differences according to Tukey’s test (α = 0.05).

Disease symptoms appeared on the tomato leaves at 21 to 35 days after inoculation with the virulent bacterial pathogen P. syringae under control conditions (Fig. 7A, 1a–1h). Symptoms included the yellowing, flaccidity, and drooping of leaves followed by necrosis and discoloration (Fig. 7A, 1a–1h). The disease severity caused by P. syringae was observed to be maximum in PCZ+P. syringae plants (68.5%) followed by 46.7% in P. syringae alone treatment (Fig. 7A and B). Reactive oxygen species (ROS) deposition indicated by the nitroblue tetrazolium (NBT) blue areas correlated with the disease incidence which was recorded highest in PCZ+P. syringae compared with that in PCZ, P. syringae alone, and control treatments (Fig. 7A). The accumulation of ROS was much weaker in W9 and W9+PCZ than that of the control, PCZ, and P. syringae groups. In the case of W9+PCZ and W9+PCZ+P. syringae, also, accumulation of O_2_^−^ was much weaker than that of the control and P. syringae alone (Fig. 7A, 2e and 2h). O_2_^−^ accumulation was decreased in PCZ+W9 compared with that in PCZ alone (Fig. 7A, 2g and 2c). The relative abundance of bacterial order related to the biocontrol of P. syringae pathogenicity differed among treatments applied to the tomato plant (Table 2). Microbial communities of these bacterial orders were negatively correlated with the disease severity on tomato plants, indicating possible biocontrol properties of some strains against bacterial speck caused by P. syringae. In particular, strong negative correlations were observed with *Bradyrhizobium* (r = −0.680), *Methylobacterium* (r = −0.650), *Janthinobacterium* (r = −0.810), Sphingomonas yabuuchi (r = −0.860), and Rhodococcus(r = −0.430) (Table 3).

**TABLE 2:** Relative abundance of endophytic bacterial orders comprising of known biocontrol agents against P. syringae

Bacterial order (genus)^*a*^	Mean relative abundance (%) of bacterial order ± SE^*b*^			
	Control	PCZ	NBRI-W9	PCZ+W9
Rhizobiales (*Methylobacterium*)	2.755 ± 0.19c	0.19 ± 0a	6.14 ± 0.36d	1.045 ± 0.24b
Sphingomonadales (*Sphingomonas*)	1.92 ± 0.08b	0.115 ± 0.01a	2.25 ± 0.26d	0.255 ± 0.08a
Actinomycetales (*Rhodococcu*s)	5.045 ± 0.11c	1.005 ± 0.06a	13.13 ± 0d	3.745 ± 0.5b

a The genus in parenthesis shows the reported genera known for biocontrol of P. syringae Methylobacterium spp. (69), Sphingomonas spp. (70), and Rhodococcus spp. (71).

b The mean values and standard errors (SEs) of three replicates are presented for each sample. For each genus, different lowercase letters indicate significant differences according to Tukey’s test (α = 0.05).

**TABLE 3:** Correlations among bacterial order and genus identified in tomato leaves with different treatments against bacterial speck disease caused by Pseudomonas syringae

Leaf endophyte community order^*b*^	Taxonomy	Correlation coefficient of relative abundance with bacterial speck disease caused by P. syringae^*a*^
*Rhizobiales* (−0.387)	*Bradyrhizobium*	**−0.68**
	*Methylobacterium*	**−0.65**
*Burkholderiales* (−0.256)	*Achromobacter*	−0.3
	*Janthinobacterium*	**−0.81**
	*Ralstonia*	−0.34
*Clostridiales* (−0.468)	Dorea formicigenerans	**0.68**
	*Ruminococcus*	0.25
*Rhodospirales* (0.684)	*Azospirillum*	**0.68**
	*Azospirillum*	0.22
*Sphingomonadales* (−0.373)	Sphingomonas eichinoids	−0.12
	*Sphingomonas yabuuchi*	**−0.86**
*Actinomycetales* (−0.366)	*Phycicoccus*	−0.29
	*Arthrobacter*	−0.32
	*Rhodococcus*	**−0.43**
*Pseudomonadales* (−0.324)	Acinetobacter	−0.098
	Pseudomonas nitroreducens	−0.098
*Lactobacillales* (0.218)	*Aerococcus*	0.26
	*Lactococcus*	0.48
*Bacillales* (0.312)	*Brevibacillus*	0.05
	*Paenibacillus*	**0.74**
	Bacillus cereus	0.15
	B. subtilis	−0.12

a Spearman’s rank correlation coefficients are based on the relative abundances of endophytic bacteria at order level in tomato leaves and the effect of microbial communities against bacterial speck disease. Significant (*P* ≤ 0.05) correlations are indicated in boldface.

b The values in parenthesis indicate the overall correlation of the order with the bacterial speck disease.

## DISCUSSION

The present study outlines the impact of fungicide and bioinoculum on the structure and dynamics of endophytes. Due to the emerging role of endophytes on plant health and protection against pathogens, their growth and sustenance are of utmost importance to promote sustainability, which is in agreement with the findings of the present study. The pesticides are well established to cause changes in nontarget microorganisms inhabiting the agroecosystem (8, 10). Applications of systemic fungicide are known to show long-term effects on the species richness and growth index of fungal communities in Vicia faba and Phaseolus vulgaris (25). However, little is known about the effects of chemical and biological plant protection strategies on the equilibrium of endophytic bacterial communities of a crop. The present study compared and revealed the impact of a biocontrol agent (B. subtilis) and a commercial fungicide (PCZ) on the endophytic bacterial community of the tomato plants and their association with the disease incidence caused by P. syringae.

An inhibitory effect of PCZ was observed on the heterogeneous bacterial populations of tomato rhizospheric soil, phyllosphere, and endophytes which corroborates with earlier reports (8, 10). The *in vitro* experiment also showed a higher sensitivity of endophytes toward PCZ than that of the phyllosphere microflora. This shows that endophyte susceptibility toward harmful chemicals was probably by virtue of its protected niche, while the phyllospheric microflora showed resistance due to its exposure to relatively harsh external conditions (26). The effect of NBRI-W9 on the overall increased endophyte population may be due to the better plant health observed in the presence of the strain. Although no studies are available, to the best of our knowledge, showing the impact of microbial inoculant application on plant endophytes, it is proposed that NBRI-W9 acts as a biostimulant which enhances the overall nutrients in the plant-providing-growth contingents to the native flora. This idea is supported by the PICRUSt analysis showing the abundance of transport- and nutrient-related metabolic pathways and also enhanced microbial activity related to chemotaxis and lipopolysaccharide production.

### Effect of W9 and PCZ on the diversity and function of tomato leaf endophytes.

An inhibitory effect of PCZ on tomato endophytes was observed *in vitro* and *in vivo* while NBRI-W9 supported the endophyte population. The marginal impact of PCZ on W9 (*in vitro*) indicates that the apparent reduction of endophyte population observed in the combined treatment was due to the loss or retarded growth of both NBRI-W9 and the natural endophytes. A permanent loss of the endophytes in PCZ strengthens the habitat loss theory due to anthropogenic activity. The SEM imaging of the phyllosphere after the treatments showed that chemical fungicide reduced the numbers of phyllosphere microflora along with morphological distortion in leaf surface compared with the control, NBRI-W9, and PCZ+W9 treatments. The external microflora reflects the endophytic bacterial diversity changes, as supported by previous reports showing that the endophytic microbiota enters plant tissues through aerial parts of the plant, such as the leaves, flowers, and fruits (27).

According to the multivariate analysis of the tomato leaf endophytes, W9 and PCZ treatments affected the overall population and composition of the endophytic bacterial communities of leaves. The results clearly demonstrate the susceptibility of the endophytic communities toward spraying of chemicals and biological agents. In particular, PCZ treatment decreased the abundance of many of the bacterial orders reported for improving plant immunity (28). Such orders like *Rhizobiales*, *Burkholderiales*, and *Actinomycetales* are also important for increasing plant resistivity to the pathogen (29–31). The increased abundance of the *Rhodospirales* in PCZ-treated plants shows the oxygen-limiting condition (hypoxia) since these groups are known to survive under anaerobic or microanaerobic conditions. *Rhodospirales* contain genera which can grow photoheterotrophically under anoxic conditions in the light, chemoheterotrophically in the dark, and heterotrophically under aerobic and microaerobic conditions (32). Hypoxic conditions are a common feature in plants experiencing abiotic and biotic stress conditions. Enhancement of these endophytic bacterial orders in the W9 treatment strengthens the utility of microbial inoculants in agriculture as reported previously (28). The marginal impact of PCZ on W9 (*in vitro*) indicates that the apparent reduction of the endophyte population observed in the combined treatment was due to the loss or retarded growth of both W9 and the natural endophytes. The strain NBRI-W9 is itself a putative endophyte of Piper chaba (22), and as expected of a true endophyte, it ought to be noninvasive; that is, it should not be hyperproliferating and interfering with the native microflora, which are characteristics of a pathogen. Based on the diversity indices, we may argue that NBRI-W9 did not show any advantageous growth over the native endophytes. This conclusion was clearly evident from the heat map showing a similar pattern of the *Bacilli* class, besides the pie chart showing a greater relative abundance of the *Bacillus* in PCZ and PCZ+W9 than that in the control and W9 treatments. Thus, typical of the chemicals, the PCZ, a fungicide, decreased the nontarget bacterial endophyte number and diversity which was ameliorated in the presence of NBRI-W9. All the more, NBRI-W9 increased the potential of the plant to harbor a greater number of endophytes and support more diverse microbes than that of the control and PCZ-treated plants. The rise in the abundance of Acinetobacter and *Arthrobacter* by about 3% in a PCZ-sprayed plant may be designated the indicators to denote PCZ contamination. Acinetobacter members are reported to degrade many different pesticides, such as permethrin, atrazine, malathion, imazamox, deltamethrin, α-endosulfan, and α-cypermethrin (33, 34). On the other hand, *Phycicoccus* appeared as an interesting genus that was greatly enhanced in NBRI-W9 treatment compared with the control (0.4%) and remained nearly undetectable in others. First proposed in year 2006, the genus is appearing to be ubiquitously present and is increasingly being reported from plants as endophytes (35, 36). However, their role as an endophyte is not known and needs to be explored.

PICRUSt gives an idea of the functional aspects of the microflora and is frequently used as a tool to understand the metabolic potential of the system. In the present study, PICRUSt observations were supported by the microbial composition and biocontrol activities. The observations from the analysis suggest that the endophytes participated in various physiological processes of the plant showing a dynamic interaction and an exchange of nutrients and other compounds which are in concurrence with the observed diversity. The type VI secretion system (T6SS) metabolic pathway gene family is reported for its presence in plant-associated bacteria and as inducers of plant immunity (37, 38). The T6SS higher copy number in W9 treatment than that the control, PCZ, and PCZ+W9 also corresponds with the higher relative abundance of proteobacteria, which are reported for having T6SSs (39). The oxidative stress caused by biotic and abiotic agents are countered by the oxidative stress resistance gene family, which was also higher in copy number in NBRI-W9 treatment. These observations are similar to the earlier reports showing a reduction of oxidative damage caused by drought stress in tomato (40). Chemotaxis-related traits were also higher in W9-treated plants in the present study which indicated active colonization (41–43) of the leaves by the endophyte, whereas the chemical fungicide showed a comparatively negative impact on the function. The higher copy number of mineral nutrition-related metabolic pathway genes like for the zinc, nitrogen, iron, and phosphate transport pathway in the presence of NBRI-W9 treatment while the low copy number in the presence of chemical fungicide show that it affected the dynamic interaction of the endophytes with the plants.

### Pathogenesis-related response of W9- and PCZ-treated tomato plants toward P. syringae.

The strain NBRI-W9 is a potent biocontrol agent of fungal pathogens, and it shows no inhibitory effect on P. syringae under *in vitro* conditions; similarly, no inhibitory effect of PCZ was observed on P. syringae. Therefore, it may be acknowledged that the pathogenesis response in the treatments involved the innate immunity status of the plant vis-a-vis the endophytic community. The potential of endophytes to suppress phytopathogens via antagonistic activity is known (44). Therefore, the endophytic community may be regarded as containing beneficial symbionts which confer a barrier effect against the pathogens in plants, similar to the gut microbiota of the humans (45, 46). Reactive oxygen species (ROS) is an oxidative stress marker and is antimicrobial in nature. Its need arises during biotic and abiotic stress in plants. The status of ROS was high in the treatments containing P. syringae, PCZ, or both, whereas in all the W9 treatments, it was lower. This finding clearly indicates that all the W9-containing treatments experienced relatively less ROS stress than the PCZ and P. syringae. While the necrotrophic effect of P. syringae explains the enhanced ROS, its high level in PCZ is debatable; disruption of the endophytes by the PCZ could be one of the reasons. The relatively unchanged population of PCZ+W9 compared with the control supports the role of endophytes and W9 during P. syringae infection (PCZ+W9+P. syringae) showing controlled ROS. In spite of reduced ROS stress, the NBRI-W9+P. syringae treatment exhibited 24% of bacterial speck disease incidence compared with 46% in P. syringae and 68% in PCZ+P. syringae. The decreased disease severity in the treatment was negatively correlated with the relative abundances of endophytic bacterial genera (*Bradyrhizobium*, *Methylobacterium*, *Janthinobacterium*, *Sphingomonas*, *Rhodococcus*, *Bacillus*, and Pseudomonas), suggesting that some strains of these genera could have a role as biocontrol agents. Thus, beneficial communities have been seen as potential plant probiotic agents (47), which could defend the host and promote its growth. In agreement with the 16S rRNA microbial community analysis results, a functional analysis of leaf communities against bacterial speck disease revealed the negative and positive impact on the endophytic bacterial communities which are modulated after treatment with the fungicide or the biocontrol agent. Furthermore, the system-level analysis of the complex interaction that governs outcomes among community members in the context of the plant host is required to identify beneficial interactions and selection processes for effective communities under specific environmental conditions and pathogen pressures. In the present study, we attempted to link functional traits (biocontrol against P. syringae) of endophytic bacterial communities with 16S rRNA bacterial diversity data of these populations and identified effective endophytic microbial communities, which may represent a new tool for crop protection. In particular, indigenous microbial communities could be stimulated with agronomic practices to restore the beneficial microbiota for plant defense.

### Conclusion.

The endophytes play a vital role in plant health; however, little information has been established about their interaction with direct and indirect external factors. The present study surmises that the application of a systemic fungicide has a negative influence on the abundance and phylogenic diversity of the bacterial endophyte community and affects their plant protective activities. The study concludes that the application of the systemic fungicide propiconazole affects the nontarget bacterial endophyte diversity and functions, compromising plant health. Alternately, a microbial inoculant enhanced the plant’s capacity to harbor a rich bacterial endophyte population and diversity which transmutes into plant immunity. The observations are important to highlight the implications of systemic pesticide applications on the endophytic niche, of which the consequences could be far reaching as the plant’s immunity is compromised. However, the microbial inoculums provide a sustainable alternative for reducing this chemical burden and provide sustainable disease resistance in plants.

## MATERIALS AND METHODS

### Experimental design.

This study was conducted to show the impact of chemical (propiconazole) and biological (Bacillus subtilis NBRI-W9) fungicides on the nontarget endophytic microbes of tomato and their implication on plant immunity. Initially, the *in vitro* experiments were conducted to see the inhibitory effect of the chemical fungicide on the natural microflora associated with tomato plants. Next, compatibility of NBRI-W9 with PCZ and the natural microflora of tomato were shown. To assess the impact of PCZ and NBRI-W9 on the bacterial endophytes of tomato, their CFU counts were determined at an interval of 24 h for 7 days. From this experiment, we also determined the time of sampling to carry out the detailed study of the bacterial endophytes by sequencing the bacterial V3-V4 region. The diversity and abundance of microbes were also associated with the plant immune response against Pseudomonas syringae which causes bacterial speck disease in tomato. The bacterial pathogen was selected as it was a nontarget organism for both PCZ and NBRI-W9, and therefore, its control would be linked directly with the plant immune status. The microbes used in the study are deposited in the type culture collection (MTCC, Chandigarh, India).

**(i) Plant material.** Tomato cultivar S22 seeds were germinated in garden soil, and 21-day-old seedlings were transplanted in pots containing 5 kg unsterilized garden soil.

**(ii) Chemical fungicide.** Commercial-grade propiconazole (PCZ) (25% emulsifiable concentration of propiconazole; Syngenta India limited, Lucknow, India) was used in the present study.

**(iii) Bacterial cultures.** Bacillus subtilis NBRI-W9 (W9), MTCC-25374, was used as a biocontrol agent (22), and P. syringae pv. maculicola ES4326 was used as the virulent pathogen in tomato.

### Compatibility of propiconazole (PCZ) with Bacillus subtilis.

The effect of PCZ on W9 growth was evaluated by the agar diffusion method (48). PCZ with 0.1% and 1% was prepared in water. The NBRI-W9 strain was cultured in nutrient broth (NB) tubes, and the culture was adjusted to approximately 1 × 10^5^ CFU mL^−1^. The culture was spread onto nutrient agar (NA) plates. A sterile 6-mm cork-borer was used to make wells on each plate. Subsequently, 100 μL of the NBRI-W9 culture was poured in agar wells, and plates were incubated at 37°C for 24 h. Streptomycin (100 μg) and water (100 μL) were used as positive and negative controls, respectively. Assays were carried out in triplicate.

### Effect of PCZ on the bacterial population of tomato rhizospheric soil, leaf phyllosphere, and endophytes.

The agar well diffusion method was used to screen the effect of PCZ (0.1% and 1%) on the microbial population of soil, phyllosphere, and endophyte of tomato leaf (49). One gram each of tomato rhizospheric soil and surface sterilized and unsterilized leaf samples was used. For soil, a 1-g sample was suspended in 10 mL saline, vortexed, and allowed to settle. The supernatant (100 μL) was spread onto NA plates having well treatments as described for the compatibility assay. Similarly,100 μL of the samples was used for phyllosphere and endophyte microbes using unsterilized and surface-sterilized leaves, respectively. Leaf samples were crushed in 1 mL saline, incubated overnight at 28 ± 2°C for enrichment, and used for spreading onto modified tryptic soya agar (TSA) (50). Modified TSA composition per L included the following: 15 g agar, 1.5 g casein peptone (pancreatic), 0.5 g sodium chloride, and 0.5 g soya peptone (papainic). The appearance of inhibition zones (including the wells diameter) was observed after 24 to 48 h of incubation. Streptomycin and water were used as the positive and negative controls, respectively.

### Determination of endophytic bacterial population at different time intervals.

Green house experiments were carried out in pots containing 5 kg unsterilized garden soil. Four-leaf-stage tomato seedlings were transplanted in the pots (1 seedling/pot). Three weeks after transplantation, the treatments were given which included control (C), 0.1% propiconazole (PCZ), W9, and PCZ+W9 and were arranged in four rows of six replicates each. NBRI-W9 was first sprayed on the plants in treatments W9 and PCZ+W9. The W9 culture was sprayed until drenching and was allowed to stabilize for 24 h. After 24 h of W9 spraying, the 0.1% PCZ was sprayed on tomato leaves in PCZ and W9+PCZ treatment plants and considered day 0 (initial day). The control was sprayed with water. For the application of W9, a bacterial suspension was prepared by growing the cultures on nutrient agar (NA) for 72 h at 28°C. The bacterial growth of W9 was scrapped in sterilized 0.85% saline, and the CFU count was adjusted to approximately 1 × 10^5^ CFU mL^−1^. For the determination of the endophyte population, the fourth leaves from the top (51), fully expanded, without any disease symptoms, were selected from the plants. The microbial count of the leaf endophytes was determined at 0, 24, 48, 72, 96, 120, and 144 h using 1 g of surface sterilized leaf samples. Leaves were washed thoroughly with sterile distilled water (SDW), immersed in 70% ethanol for 30 to 45 s, and rinsed with SDW. The leaves were then dipped in a 3% sodium hypochlorite solution for 3 min followed by 5 to 7 times of thorough washing with SDW. The final portion of the SDW washing was spread onto an NA plate to ensure surface sterilization of the leaves. The leaves were crushed in 1 mL saline, serially diluted, spread onto modified TSA media (50), and incubated at room temperature for 48 h at 30°C. The number of bacterial colonies was noted, and CFU was calculated in terms of log_10_ CFU gm^−1^.

**(i) Microscopy.** The leaf surface microbiota was studied after application of the treatments using scanning electron microscopy (SEM). For SEM studies, the samples were prepared using fresh tomato leaf samples taken at 72 h of (bio)fungicide application. The tissues were fixed overnight in 2.0% (vol/vol) glutaraldehyde in 0.08 M sodium phosphate buffer (pH 7.2), rinsed three times for 5 min in 0.1 M sodium phosphate buffer, dehydrated in ascending concentrations of ethanol (30, 50, 70, 95, and 100% [vol/vol]) for 15 min at each concentration, and then dried in a Samdri-PVT-3D critical point dryer (Tousimis, Rockville, MD). Dehydrated leaf samples were mounted on aluminum stubs (Ted Pella Inc., Redding, CA) using double-sided carbon tape (Ted Pella Inc.) and were gold coated for 60 s in a Pelco Auto Sputter Coater SC-7 (Ted Pella Inc.). SEM images were taken using a Phenom Pro (Phenom-World, Eindhoven, The Netherlands) with a 5-kV accelerating voltage.

### Endophytic bacterial community analysis.

A pot experiment was repeated under greenhouse conditions as described above. To analyze bacterial diversity, leaves were collected after 72 h of PCZ treatment. Leaves were collected from six different replicates, surface sterilized, frozen in liquid N_2_, and processed for 16S rRNA microbial diversity analysis.

**(i) DNA extraction and amplification.** DNA was extracted from surface-sterilized tomato leaves using a genomic DNA isolation kit according to the manufacturer’s instructions. The 16S rRNA gene amplicons were amplified following the 16S rRNA gene sequencing library preparation Illumina protocol. The gene-specific sequences used in this protocol target the V3 and V4 region of 16S rRNA gene (52). Illumina adapter overhang nucleotide sequences were added to the gene-specific sequences. The primer pair included a forward primer (5′TCGTCGGCAGCGTCAGATGTGTATAAGAGACAGCCTACGGGNGGCWGCAG-3′) and reverse primer (5′GTCTCGTGGGCTCGGAGATGTGTATAAGAGACAGGACTACHVGGGTATCTAATCC 3′). A multiplexing step was performed using Nextera XT index kit (FC-131-1096). A total of 1 μL of the PCR product was run on a bioanalyzer DNA 1000 chip to verify the size; the expected size on a bioanalyzer trace should be ~550 bp. The libraries were sequenced using a 2 × 300-bp paired-end run on a MiSeq sequencer according to manufacturer’s instructions (Illumina).

**(ii) Bioinformatics analysis and data processing.** The quality of the sequencing reads was checked through FastQC (53). Raw reads undergo a strict filtering process (53) to obtain high-quality data according to the QIIME (54) quality-control process. Paired-end reads were filtered to eliminate the low-quality reads via quality filter using Trimmomatic 0.36 with rigorous filtering criteria. Chloroplast OTUs as well as nonidentified OTUs were removed. Further reads were processed using QIIME (v1.9.0) (55). The effective sequences were grouped into operational taxonomic units (OTUs) against the Greengenes v13.5 database (56). An OTU-based analysis was performed to calculate the richness, diversity, and evenness at 97% sequence similarity coverage. The representative sequence of OTUs was selected. The microbial community structure and diversity among samples were calculated using the QIIME pipeline with a Bray-Curtis dissimilarity matrix (57). The results were displayed using principal-coordinate analysis (PCoA) (58) and plotted with the vegan R package. Alpha diversity metrics were calculated by using random subsampling of the OTU table in the QIIME pipeline. Furthermore, rarefaction curves were obtained for each sample using vegan R package. The taxonomic classification was done with the KAIJU program (59) based on the clean reads, and furthermore, these classifications were viewed via a KRONA (60) plot. Multivariate analyses were performed on a Bray-Curtis resemblance matrix of square-root-transformed relative abundances, and principal-component analysis (PCA) was also performed. The hierarchal clustering analysis was performed by using Calypso (61). The diversity within each sample was estimated using the Shannon diversity index (62). Microbiome profiling at the family level drawn based on a database of taxon-specific marker genes was performed using MetaPhlAn2 (63) with the Spearman algorithm. The outcomes of MetaPhlAn2 analyses were plotted through GraPhlAn (64). The relative taxonomic abundance of the taxa was inferred by the vegan R package. Moreover, hierarchical clustering analysis across different variables within four samples was done using Pearson’s correlation.

**(iii) Phylogenetic investigation of communities by reconstruction of unobserved states (PICRUSt) analysis.** A PICRUSt analysis was performed to predict the microbial community functional content based on the software package (PICRUSt v1.0.0) (65). This approach exploits the relationship between phylogeny and function by combining 16S data with a database of reference genomes (Greengenes) to predict the presence of gene families. The 16S rRNA sequences were clustered into a collection of OTUs using a closed reference OTU picking protocol (QIIME 1.8.0) (55). The obtained OTU table was normalized by 16S rRNA gene copy number and then used to predict microbial community functional content based on the PICRUSt software package (65). Functional predictions were exported as KEGG orthologs.

### Correlation analysis of leaf endophytic microbiota and disease severity caused by Pseudomonas syringae in tomato plants with pathogenicity assay and histochemical staining.

**(i) Pathogenicity assay of P. syringae.** The susceptibility of PCZ- and W9-treated tomato plants toward P. syringae pv. maculicola ES4326 was studied under greenhouse conditions in pots containing garden soil at 25°C with a 12-h light (100 μE m^−2^ s^−1^)/12-h dark cycle and a relative humidity of 60% ± 10%. The treatments included the following: (i) control, (ii) P. syringae, (iii) PCZ+P. syringae, (iv) W9+P. syringae, and (v) PCZ+W9+P. syringae. The treatments were given on 1-month-old plants, as described in earlier experiments. Initially tomato seedlings were treated with NBRI-W9 (10^5^CFU·mL^−1^) and PCZ (0.1%) for 3 consecutive weeks (once a week) by spray inoculation. Then, P. syringae was sprayed 1 week after the third spray of NBRI-W9 and PCZ treatments to ensure no direct/immediate effects of the W9 and PCZ and to get real picture of the plant immunity. For application of P. syringae, the bacterial suspension was prepared by growing the cultures on King’s B media for 72 h at 28°C. The CFU of P. syringae was adjusted to approximately 1 × 10^7^ CFU mL^−1^ for spray application using a hand sprayer until drenching. The pathogen was applied three times (i.e., once in a week) to ensure pathogenesis. The SDW was applied as the mock inoculation in control plants. Symptoms were noted 2 weeks after the final pathogen spray. Disease severity was evaluated visually by determining the percentage of diseased leaves and scoring the specks following an index of 0 = no symptoms, 1 = 2 to 5 specks together or spread all over the leaf, 2 = 6 to 10 specks, and 3 = more than 11 specks (66, 67). The formula used to convert the scores in terms of percent disease severity was (sum of disease index)/(total leaves assessed) × 100. A total of 16 leaves per treatment was used for scoring.

**(ii) Histochemical staining of fungicide-treated tomato plant leaf.** Reactive oxygen species was detected using the nitroblue tetrazolium (NBT) staining method described previously (68) to determine the accumulation of O^2−^ in the different treatments given to the tomato plants. In brief, the entire leaflet was harvested at the end of the subjective photoperiod on day 21 and submerged in 10 mL of 50 mM sodium phosphate buffer (pH 7.5) containing 2 mg⋅mL^−1^ NBT (Thermo Fisher Scientific Inc., USA). It was then placed under a vacuum for 5 min and incubated at 45 min. A set of leaflets was incubated in a solution of 50 mM sodium phosphate buffer (pH 7.5) without NBT for use as a negative control. Subsequently, the leaflet was bleached by boiling twice in bleaching solution (ethanol-acetic acid-glycerol, 3:1:1 [vol/vol/vol]) for 15 min each. The image of the stained leaflet was obtained on a black background using the Nikon digital camera D5200.

### Statistical analysis.

The data of the PCZ effect on the natural bacterial population and microbial relative abundance were analyzed using IBM SPSS statistics software 20. After validation of a normal distribution (Kolmogorov-Smirnov [K-S] test, *P* > 0.05) and variance homogeneity (Levene’s test, *P* > 0.05) of the data, analysis of variance (ANOVA) was carried out using Tukey’s test (α ≤ 0.05) to detect significant differences among treatments. Relative abundance data were normalized by root square transformation. To detect significant differences in microbial abundances among treatments, ANOVA was applied using Tukey’s test (α ≤ 0.05).

### Data availability.

Sequencing data were deposited in the Sequence Read Archive (https://submit.ncbi.nlm.nih.gov/subs/sra/) and NCBI under accession numbers SRR14844087, SRR14844085, SRR14844086, and SRR14844084 and BioProject number PRJNA738668.
